# Immunogenicity of Anti-TNF-α Biotherapies: II. Clinical Relevance of Methods Used for Anti-Drug Antibody Detection

**DOI:** 10.3389/fimmu.2015.00109

**Published:** 2015-04-08

**Authors:** Klaus Bendtzen

**Affiliations:** ^1^Institute for Inflammation Research (IIR 7521), Rigshospitalet University Hospital, Copenhagen, Denmark

**Keywords:** anti-TNF biopharmaceuticals, immunogenicity, anti-drug antibodies, enzyme-linked immunosorbent assay, homogeneous mobility-shift assay, cell-based reporter-gene assay

## Abstract

Immunogenicity of biopharmaceuticals is complex and influenced by both structural and pharmacological factors, and by patient-related conditions such as disease being treated, previous and concomitant therapies, and individual immune responsiveness. Essential for tailored therapeutic strategies based on immunopharmacological evidence from individual patients (personalized medicine) is the use of assays for anti-drug antibodies (ADA) that are accurate and relevant in the clinical setting. This paper discusses immunogenicity of genetically engineered immunoglobulins directed against tumor-necrosis factor-α (TNF). Emphasis will be on commonly used methods for detection of ADA in human serum including issues that question the clinical applicability of these methodologies. The use of dubious assays for ADA in a clinical context may not only contribute to confusion as to the importance of drug immunogenicity but may also prevent development of safe and cost-effective ways of using biological TNF-antagonists.

## Introduction

**Figure 1:**
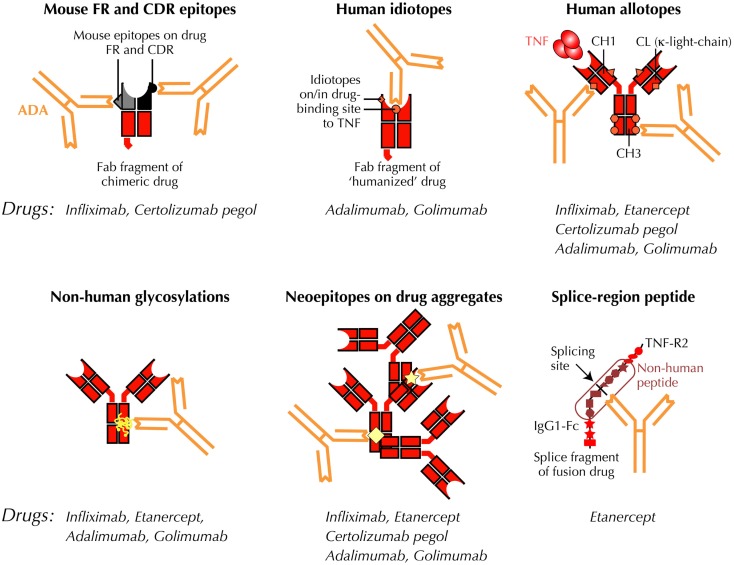
**Putative immunogenic sites on anti-TNF antibody constructs**. Antibody constructs, drugs, and drug fragments with “human” aminoacid sequences, are depicted in red. Mouse sequences are shown in black/gray. ADA, anti-drug antibody; CDR, complementarity- determining variable region of antibody; CH1, CH3, CL, constant regions of IgG on light- and heavy-chains, respectively; Fab, antigen-binding region of antibody; Fc, crystallizable region of antibody; FR, framework region of antibody; TNF, tumor-necrosis factor; TNF-R2, TNF type 2, p75 receptor; VH, VL, variable regions of IgG on heavy and light chains, respectively.

Immunogenicity is a risk associated with all genetically engineered proteins, and repeated injections of humanized biopharmaceuticals may generate anti-drug antibodies (ADA), which can be related to drug failure and side effects (1). Examples are swine and human insulin, growth hormone, factor VIII, factor IX, erythropoietin, type I interferons, and a host of more or less “humanized” antibody constructs (2). The latter include biopharmaceuticals that target the inflammatory cytokine, tumor-necrosis factor-α (TNF) (Figure 1) (3–5). It has, for example, been documented repeatedly that the appearance of ADA against biological TNF-antagonists is a frequent occurrence, and that this is closely associated with disappearance of drug in the circulation, and response failure (2, 6, 7).

The importance of individualization of therapies with protein drugs has been increasingly recognized in recent years (7). This is particularly important with administration of costly and widely used biological drugs, where investigations suggest that therapeutic decision making should be based on immunopharmacological monitoring in addition to clinical outcome. A rational, but frequently overlooked approach to accomplish this would be the use of reliable and clinically relevant methods for ADA (and drug) detection in biological fluids.

This paper briefly describes commonly used assays for circulating ADA with focus on applicability as clinical tools to improve anti-TNF therapies, including cost-effectiveness.

## Neutralizing and Non-Neutralizing ADA

Neutralizing ADA directly interferes with the ability of biological TNF-inhibitors to block TNF signaling through specific TNF-receptors on target cells. These ADA may be directed against idiotopes in (or outside) the TNF-binding fragments (Fab) of the anti-TNF immunoglobulin construct (Figure 1). Depending on binding characteristics such as affinity and association/dissociation kinetics, these anti-idiotypic antibodies may directly prevent a drug from binding TNF. Neutralizing ADA may, however, also result from ADA binding to other sites on the drugs, for example, if binding of one or more ADA molecules result in steric changes that prevent a drug from attaching to TNF, or if ADA-binding results in drug aggregation and/or immune complex formation that masks TNF-binding sites on individual drug molecules.

Neutralizing ADA is generally thought to be more important in the clinical setting than non-neutralizing ADA. The latter, however, may indirectly reduce therapeutic efficacy by compromising bioavailability and/or accelerate drug clearance from the circulation. Thus, in cases where TNF-antagonists are administered subcutaneously, non-neutralizing (as well as neutralizing) ADA may form immune complexes around injection sites reducing drug transfer to the circulation. Non-neutralizing ADA may also alter the pharmacokinetics (PK) of drugs influencing tissue availability of TNF-antagonists through formation of immune complexes and subsequent removal of drug from the circulation through endothelial impact, spleen filtration, binding to Fcγ receptors on phagocytic cells, and Brambell receptor-mediated recycling. All of these processes are likely to be independent of the drug’s ability to bind TNF, as are side effects caused by drug – ADA complexes.

## Trough Level ADA Assessments

The conventional approach to test for ADA is to assess serum samples collected at the end of a therapeutic cycle (trough levels). This originates from the fact that almost all commonly used assays are drug-sensitive, so that they cannot accurately detect ADA in blood collected closer to drug administration (8). Unfortunately, measuring trough levels of ADA limits the clinical usefulness of the test results. This is because *trough* is often ill-defined. Drug holidays, for example, are sometimes needed in patients with intercurrent diseases, or just for practical reasons. Assessing trough levels of ADA under these circumstances, i.e., long after drug administration, may result in higher than “normal” levels, particularly if immunization has progressed from a primary immune response to a prolonged and more potent secondary response. Therapeutic failure may also lead to trials with shortened intervals of drug administration, which would result in lower than “normal” trough levels of ADA due to rapid removal from the circulation of newly formed drug – ADA complexes.

Assessment of immunogenicity of TNF-antagonists is also affected by different dosing intervals. Etanercept, for example, is administered once weekly, and this frequent administration results in high drug levels even in trough samples, making it difficult for a drug-sensitive test to reveal the presence of anti-etanercept ADA.

An approach to overcome this problem would be to separate drug – antibody complexes before or during the assay. This may be accomplished by acid dissociation of immune complexes (9). In a variant of this assay, adapted for detection of ADA against adalimumab, the immune complexes are dissociated by adding acid and rabbit anti-idiotype-F(ab) (10). The rabbit F(ab) fragments inhibit reformation of ADA – drug complexes by competing with ADA for drug binding. Released ADA is then measured by an antigen-binding radioimmunoassay. Unfortunately, these assays are laborious and difficult to adapt to routine use if carried out by radioimmunoassay. Incomplete dissociation of the immune complexes and/or reassociation before completion of the assay are other potential problems. The process of pH-shifting during testing may also introduce artifacts that are difficult to control, including irreversible destruction of ADA-binding epitopes on drug molecules *in vivo*.

Another approach would be to develop techniques for more robust PK analyses instead of those deducted from PK surrogates in trough serum samples. This is now possible with the development of reporter-gene assays (RGA) that monitor TNF-mediated activation of TNF-receptor-bearing target cells; see below. This enables detection of TNF neutralization (provided by the drug) and neutralization of this effect (by ADA). Such a cell-based assay would mirror processes at the cellular level *in vivo*, and it might be used to assess anti-TNF activities in the blood at drug delivery and at various times thereafter. It is, for example, likely that peak TNF-inhibitory activity in a therapeutic cycle would provide more information of clinical relevance than trough drug and ADA levels.

## Problems with Currently Used Assays for ADA

**Figure 2:**
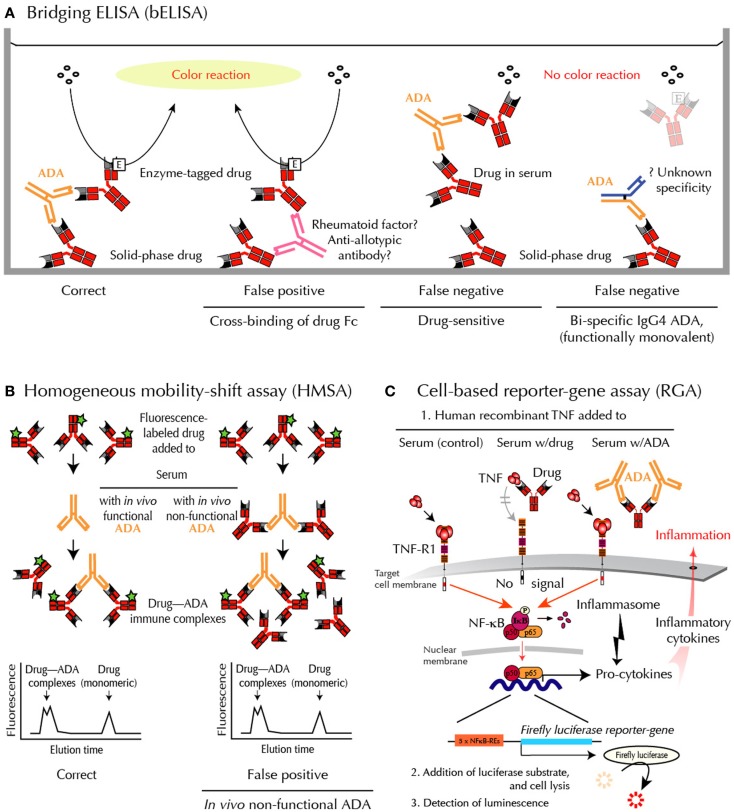
**Methods for ADA detection – and shortcomings**. **(A)** bELISA for drug-binding ADA. bELISA depends on the bivalency of IgG ADA (and multivalency of IgA and IgM ADA) and therefore the ability of these immunoglobulins to “bridge” drug molecules preadsorbed to a plastic well with an added enzyme-labeled drug molecule (left panel). Note that IgG4 antibodies are usually bispecific because half molecules are exchanged after synthesis. They are therefore “invisible” in bELISA (right panel). **(B)** HMSA for drug-binding ADA. HMSA depends on association of fluorescence-labeled drug added to serum and subsequent chromatographic separation of ADA-bound and free tagged drug (left panel). Note that functionally inactive ADA, bound to drug *in vivo*, may be split during assay and reassociated with tagged drug before or during chromatography (right panel), thus reporting similar data as visualized in the left panel. **(C)** RGA for neutralizing ADA. RGA reports functional levels of all classes of drug-neutralizing ADA and, in addition, functional levels of all currently used anti-TNF drugs. When human recombinant TNF is added to the target cells, the cytokine initiates intracellular signaling through the surface TNF-receptor, type 1 (TNF-R1), thus activating the cytoplasmic nuclear factor (NF)-κB. The active components of this transcription factor are then transported into the nucleus where they bind to NF-κB response elements (NF-κB-REs) in the genome. This activates more than a hundred genes, including an inserted reporter-gene construct encoding the enzyme Firefly luciferase. After cell lysis and addition of substrate, luciferase-catalyzed light emission can be quantified. When TNF is preincubated with patient serum containing an anti-TNF drug and then added to the cells (step 1 mid), the drug, if functional, neutralizes the effect of TNF, and no intracellular signal is initiated. When TNF is preincubated with patient serum containing drug-neutralizing ADA and then added to the cells (step 1 right), the drug no longer interferes with TNF-mediated signaling, resulting in a luminescence signal.

Assessing ADA is especially difficult when testing binding of ADA directed against drugs that are also antibodies. In these cases, standard laboratory techniques may fail to provide accurate and clinically useful results (8). The most commonly used assays include various modifications of the enzyme-linked immunosorbent assay (ELISA). Unfortunately, however, rheumatoid factors, anti-allotypic antibodies, and heterophilic antibodies may interfere with readout in these assays. In addition, the frequently used bridging-type ELISA (bELISA) fail to detect IgG4 ADA, which may dominate after prolonged immunizations (Figure 2). Finally, all solid-phase techniques are sensitive to artifacts such as epitope shielding and neoepitope formation because protein drugs, including anti-TNF biopharmaceuticals, may aggregate on plastic surfaces (7).

Less artificial fluid-phase techniques, for example, certain radioimmunoassays, better reflect the *in vivo* conditions and are therefore considered more robust in the clinical setting providing fewer false-negative and false-positive results, which is essential when an assay is used for individual therapeutic guidance (5–7).

A shortcoming of all binding assays is that they do not distinguish between inactive (non-neutralizing) and functionally active (neutralizing) ADA. This is essential for a more precise understanding of why therapies fail in some patients and not in others, as recognized by regulatory authorities^1^. For example, routine binding assays do not inform about binding kinetics and whether or not an observed attachment between drug and ADA is capable of reducing the drug’s ability to compete with high-affinity cellular TNF-receptors in a manner that prevents TNF-induced signaling *in vivo*. Another shortcoming of binding assays for ADA is the lab-to-lab variations between these techniques. This underscores the requirement for universal ADA standards when comparing binding-data from different laboratories.

With this in mind and realizing the artificial setup of most solid-phase techniques in general, it is plausible that the wide use of these assays for ADA detection have contributed to the current uncertainty surrounding drug immunogenicity. Unfortunately, this confusion may continue if clinical relevance of technologies used for therapeutic monitoring is ignored. Newly developed solid-phase binding assays, including those based on chip and bead technologies, may have similar draw-backs as they report antibody binding to drugs in an aggregated or otherwise denatured form.

## Assays for Binding ADA

Enzyme-linked immunosorbent assays are the most commonly used tests for ADA in patient serum. There are two major variations, sandwich ELISA and bELISA:

1. Sandwich ELISA detects ADA in serum by their ability to bind to plastic-immobilized Fab or Fab2 fragments of the antibody-derived anti-TNF drug. This assay is affordable and easy to use. It is, however, a potential problem that binding takes place to a drug attached to a plastic surface in a more or less aggregated form, as this increases the risk of neoepitope formation and epitope shielding (11). False-positive results have been observed if washing procedures are insufficient during testings, as this enables “sticky” Fc-fragments of antibodies to cross-bind to immobilized antibody-derived drug irrespective of specificities (11).
2. The bELISA modification is shown in Figure 2A. This assay depends on the bivalency or multivalency of all major immunoglobulin classes of ADA. It is sensitive and relatively easy to setup and use. Unfortunately, however, this methodology has significant draw-backs when used in the clinical setting. False-positive ADA results may, for example, arise from cross-binding of drug Fc-fragments by rheumatoid factor, anti-allotypic antibodies, and/or low-affinity antibodies, including heterophilic antibodies in patient sera. This technology also fails to detect IgG4 ADA, an IgG isotype that dominates after prolonged immunizations (12, 13). This is because IgG4 antibodies are functionally monovalent and therefore cannot “bridge” in this type of binding assay (Figure 2A). More importantly for accurate patient monitoring is the fact that bELISA is highly drug-sensitive with a risk of false-negative findings (7, 8). Some investigators report ADA status as “inconclusive” if drug is detectable in sera testing negative for ADA using bELISA. This has been estimated to be the case in up to half the patients in the clinical setting.

In addition to the above shortcomings, all solid-phase assays have several noteworthy limitations. These include difficulties for capture ELISA to detect anti-idiotypic antibodies because idiotopes in the TNF-binding site(s) of biological TNF-antagonists are concealed by plastic-immobilized TNF in the capture phase of the assay (7).

Homogeneous mobility-shift assay (HMSA) uses size exclusion high-performance liquid chromatography to determine serum levels of ADA (Figure 2B). This technology has been introduced in North America as a replacement for ELISA (14). The clinical usefulness of HMSA is currently investigated, but its expensive setup may limit routine use. It is a potential problem that immune complexes may be artificially split during chromatography. This will report antibodies that are non-neutralizing *in vivo* because they circulate as drug – ADA immune complexes. A recent study supports this, as the majority of HMSA-reported ADA in infliximab-treated patients was functionally inactive judged by parallel testings for neutralizing ADA (5).

## Cell-Based Assays for Neutralizing ADA

If an appropriate assay is available, regulatory authorities recommend that cell-based assays be used to quantify neutralizing ADA against therapeutic proteins^2^. In the case of neutralizing ADA against TNF-antagonists, such assays are usually based on the ability of TNF to kill susceptible cell lines. These assays are, however, difficult to standardize, take days to complete, are subject to serum matrix effects, and require cell-growth facilities. They are also limited by the fact that factors in patient sera may interfere with the assay outcome.

Reporter-gene assay is the most recent development in the efforts to assess ADA against TNF-inhibitors in a clinical context (15) (Figure 2C). It is a cell-based assay, which does not have the same characteristics as common binding assays such as ELISA and HMSA. Unlike these assays, RGA detects TNF activity, not drug or ADA *sui generis* (7). Rather, it gives a functional assessment of biologically active drug counteracted by ADA, but only if the latter bind with sufficient avidity to a locality (epitope) on the drug that enables interference with TNF-R-mediated intracellular signaling (neutralizing ADA). This closely resembles the conditions under which TNF-antagonists are believed to function *in vivo*.

Known limitations of cell-based assays have been overcome by construction of an internally normalized RGA that allows quantifications independent of serum matrix effects and cell-numbers (15). The use of assay-ready cells stored at −80°C also obviates the need for cell cultivation. Though less sensitive than certain binding assays, notably radioimmunoassay, and HMSA, RGA performs well in the clinical setting (5). It is highly specific for TNF, and is easily modified to monitor patients treated with all known anti-TNF biopharmaceuticals.

## Conclusion and Perspective

- Most biological TNF-antagonists are immunogenic even if claimed to be “fully human.”
- A prerequisite for rational long-term use of these drugs is guidance by accurate assessments of functional ADA (and drug) in the circulation, if not routinely then every time therapy fails or side-effects occur.
- Binding assays have important limitations in the clinical setting. This is primarily due to the artificial nature of these methods and drug-sensitivity, resulting in false-negative ADA assessments.
- ADA detected by high-sensitivity binding assays such as radioimmunoassay and HMSA are often non-neutralizing *in vivo* and therefore of questionable therapeutic relevance.
- Regulatory authorities recommend that cell-based assays be used to quantify neutralizing ADA against therapeutic proteins.

## Conflict of Interest Statement

Financial support was obtained from the Danish Biotechnology Program. Within the last 3 years, the author has received speaker fees from Pfizer and Biomonitor, and owns stocks in the latter.

## Footnotes

^1^http://www.ema.europa.eu/docs/en_GB/document_library/Scientific_guideline/2009/09/WC500003946.pdf

^2^http://www.fda.gov/downloads/Drugs/GuidanceComplianceRegulatoryInformation/Guidances/UCM192750.pdf
